# Targeting HMGB1 for Renal Ischemia and Reperfusion Injury: Mechanisms and Therapeutic Strategies

**DOI:** 10.3390/ph19091358

**Published:** 2026-08-27

**Authors:** Xiao-Hui Chi, Ming-Feng Liao, Ya-Qun Zhou

**Affiliations:** Department of Anesthesiology and Pain Medicine, Hubei Key Laboratory of Geriatric Anesthesia and Perioperative Brain Health, and Wuhan Clinical Research Center for Geriatric Anesthesia, Tongji Hospital, Tongji Medical College, Huazhong University of Science and Technology, Wuhan 430030, China; xh_chi@163.com

**Keywords:** HMGB1, renal ischemia-reperfusion injury, inflammation

## Abstract

Although the underlying mechanisms of renal IRI have been extensively studied, the corresponding effective treatments are still lacking. HMGB1, an important nuclear factor that is secreted outside cells when experiencing stress conditions, acts as a DAMP and exerts crucial effects on renal IRI. Many studies have suggested that the effect of HMGB1 on kidney damage is mediated mainly through the interaction of HMGB1 with pattern recognition receptors such as TLR4 and RAGE, which then results in the aggravation of local inflammatory response, increased infiltration of leukocytes, and finally renal tubular damage. Preclinical studies using animal models have demonstrated that inhibition of HMGB1 and downstream signal pathways can attenuate renal injury. This review critically evaluates HMGB1 in renal IRI across redox state, subcellular localization, temporal and cell-specific release, receptor usage, integrated stress pathways, autophagy, and regulated cell death. It also compares direct neutralization, inhibition of release or translocation, epigenetic/RNA-based regulation, and receptor-directed strategies. The evidence is predominantly derived from short-term rodent studies, with one large-animal antibody study and no therapeutic human trials. Accordingly, HMGB1 is best regarded as a biologically compelling but clinically unvalidated target whose therapeutic value will depend on redox- and phase-selective inhibition, kidney-directed delivery, and rigorous pharmacokinetic and safety evaluation.

## 1. Introduction

Renal ischemia-reperfusion injury (IRI) is a common and severe form of kidney damage that occurs in various clinical settings, such as kidney transplantation, trauma, and major surgery [1]. IRI involves an initial phase of ischemia, during which blood flow to the kidney is reduced or completely interrupted, followed by a reperfusion phase that restores perfusion [2,3]. Paradoxically, although reperfusion is essential for tissue recovery, it also initiates a cascade of detrimental events that aggravate kidney injury. These events include excessive inflammation, oxidative stress, apoptosis, and fibrotic remodeling, ultimately contributing to acute kidney injury (AKI) and increasing the risk of progression to chronic kidney disease (CKD) [4,5].

Pathophysiology of renal IRI involves multiple factors, including the intricate relationship between cellular and molecular aspects [6,7]. The involvement of the immune system plays a vital role in aggravating the process. High-mobility group box 1 (HMGB1) is a nuclear non-histone chromatin-binding protein that regulates DNA architecture and transcription, and it can act as an extracellular DAMP after passive release or active secretion under stress conditions. Multiple preclinical studies have highlighted HMGB1 as a potential therapeutic target in renal IRI [8,9,10,11,12,13,14]. In preclinical models, inhibition of HMGB1 release, interference with its receptor interactions, and neutralization of extracellular HMGB1 have been associated with reduced inflammation and renal injury [15,16,17,18,19,20]. These findings support further investigation of HMGB1 as a therapeutic target for renal IRI.

This review focuses on the functional involvement of HMGB1 in renal IRI, specifically in inflammation, oxidative stress, ER stress, and apoptosis. Additionally, we discuss various treatment methods currently to inhibit HMGB1 functions through neutralization, inhibition of translocation or secretion, epigenetics, or receptor antagonism. Through discussion of the various roles of HMGB1 in renal IRI, as well as potential therapies against HMGB1, we aim to provide useful insights in renal protection and regeneration.

## 2. Search Strategy and Literature Selection Criteria

This narrative review was updated through July 2026. PubMed was searched using: (HMGB1[Title/Abstract] OR “high mobility group box 1” [Title/Abstract]) AND (kidney [Title/Abstract] OR renal [Title/Abstract]) AND (“ischemia reperfusion” [Title/Abstract] OR “ischaemia reperfusion” [Title/Abstract] OR IRI [Title/Abstract] OR “acute kidney injury” [Title/Abstract]). Reference lists of eligible reports and relevant reviews were also screened. We included peer-reviewed English-language original studies directly examining HMGB1 in renal IRI or closely related AKI models, together with mechanistic HMGB1 studies required to interpret redox, receptor, autophagy, and repair biology. Duplicate reports, conference abstracts, non-peer-reviewed material, and non-renal studies without direct mechanistic relevance were excluded.

## 3. An Overview of HMGB1

HMGB1 is a highly conserved non-histone chromatin-binding protein belonging to the high-mobility group box family [21,22]. It contains two conserved DNA-binding domains, termed the A-box and B-box, which allow HMGB1 to interact with the minor groove of DNA. These domains are essential for its nuclear functions, including the regulation of DNA repair, transcription, and chromatin remodeling. In the nucleus, HMGB1 also serves as a scaffold to facilitate the binding of various transcription factors and DNA repair enzymes, thus maintaining cellular homeostasis under both physiological and stress conditions [23,24].

### 3.1. Redox State, Subcellular Localization, and Receptor Selectivity

HMGB1 is not a biologically uniform extracellular ligand. Its three cysteines (C23, C45, and C106) generate functionally distinct redox isoforms. Fully reduced (all-thiol) HMGB1 complexes with CXCL12 and signals through CXCR4 to promote chemotaxis and, in some injury models, tissue regeneration; disulfide HMGB1, containing a C23-C45 bond with reduced C106, engages MD-2/TLR4 and drives cytokine production; terminal oxidation of the cysteines largely abolishes these activities [25,26,27,28]. RAGE is an established receptor for extracellular HMGB1 and can mediate HMGB1 binding and cellular internalization [29,30], whereas TLR2 has also been implicated in HMGB1-induced innate immune activation [31]. Thus, the term HMGB1 should be interpreted according to redox state, molecular complex, and receptor context rather than as a single invariant mediator.

Importantly, the predominant redox isoform in renal IRI has not been directly established by redox-resolved mass spectrometry. The rapidly changing renal redox environment makes a mixed and time-dependent distribution plausible, but this remains an inference rather than a demonstrated fact. This uncertainty affects therapeutic design: broad neutralization may suppress pathogenic disulfide HMGB1, but could also remove reduced HMGB1-CXCL12 signals involved in cell recruitment and repair.

In pathological situations such as IRI, HMGB1 undergoes a translocation process, moving out of the nucleus and entering the cytoplasm before being secreted into the extracellular space [32,33]. Once outside the cell, HMGB1 functions as a prototypical damage-associated molecular pattern (DAMP), initiating and propagating immune responses through interaction with pattern recognition receptors on immune cells. Two of the most widely researched receptors for extracellular HMGB1 include the receptor for advanced glycation end products (RAGE) and Toll-like receptor 4 (TLR4) [34]. HMGB1 receptor binding triggers a series of signaling pathways, including NF-κB and MAPK pathways, that result in pro-inflammatory cytokine secretion and recruitment of immune cells [35].

In renal IRI, HMGB1 plays a pivotal role in initiating and amplifying the inflammatory cascade [10,13,15,36]. In the ischemia phase, HMGB1 is passively released from the necrotic renal cells, as well as actively secreted by immune cells such as macrophages and dendritic cells. Once released, HMGB1 binds to TLR4 and RAGE expressed on immune cells, activating the downstream signaling cascades, such as NF-κB and MAPK signaling pathways. These events amplify inflammation and result in renal damage. In addition to inflammation, HMGB1 has been shown to be involved in other pathological responses, such as oxidative stress and apoptosis [16,17,19,37]. The enhanced activity of immune cells induced by HMGB1 contributes to reactive oxygen species (ROS) generation and causes oxidative damage. Additionally, HMGB1 activates programmed cell death pathways, promoting cell death.

### 3.2. Spatiotemporal and Cell-Specific Sources

During the early ischemic and reperfusion phases, renal parenchymal cells constitute an important source of extracellular HMGB1. In particular, HMGB1 undergoes rapid nuclear-to-cytoplasmic translocation in tubular epithelial and vascular cells and can subsequently be released into the extracellular space and renal circulation [10,14,37]. Experimental hypoxic injury also induces HMGB1 release from tubular epithelial cells, further supporting these cells as an important source of extracellular HMGB1 during acute renal injury [10]. In parallel, neutrophils and macrophages accumulate in the postischemic kidney as components of HMGB1-dependent inflammation [38]. However, the extent to which these infiltrating immune cells themselves constitute quantitatively important secondary sources of extracellular HMGB1 during renal IRI remains unclear. The functional importance of cellular HMGB1 also changes during recovery. In cycling tubular epithelial cells, intracellular HMGB1 increases susceptibility to oxidative stress and contributes to AKI-to-CKD transition [17]. Collectively, these findings suggest that the cellular origin, subcellular localization, and biological effects of HMGB1 vary over the course of renal IRI, although their relative contributions have not yet been defined by longitudinal, cell-resolved analyses.

This spatiotemporal and subcellular compartmentalization may also help reconcile the apparently opposing functions of HMGB1 during tissue injury and repair. Nuclear HMGB1 contributes to chromatin organization and DNA repair, whereas cytoplasmic HMGB1 can promote Beclin-1-dependent adaptive autophagy [39]. In the extracellular compartment, the biological effect of HMGB1 is additionally influenced by its redox state: reduced HMGB1 can form a complex with CXCL12 and signal through CXCR4 to promote cell recruitment and tissue regeneration [25,28], whereas proinflammatory extracellular HMGB1 contributes to the amplification of innate immune responses. These context-dependent functions have important therapeutic implications. Early and transient blockade of extracellular proinflammatory HMGB1 may attenuate acute renal injury, whereas prolonged or nonselective inhibition during the recovery phase could potentially interfere with intracellular homeostatic functions or regenerative signaling. Whether such prolonged HMGB1 inhibition adversely affects epithelial repair, immune defense, or long-term renal recovery remains insufficiently investigated in renal IRI.

## 4. Mechanisms of HMGB1 in Renal IRI

### 4.1. HMGB1 and the Inflammatory Cascade

HMGB1 has been implicated as an important mediator of inflammation in renal IRI. Multiple preclinical studies support its involvement in the initiation and amplification of inflammatory responses. For instance, Rabadi et al. noted that there was a correlation between HMGB1 release and the elevation of systemic levels of pro-inflammatory cytokines such as TNF-α and IL-6, which lead to renal injury [14]. This evidence highlights the importance of HMGB1 as an important player in inflammation in IRI. Nevertheless, there has not been much discussion regarding the temporal dynamics of HMGB1 release in relation to its synergy with other mediators. Evidence from other acute sterile injury settings, including intracerebral hemorrhage, indicates that resident and infiltrating immune cells exhibit marked temporal and functional heterogeneity, with distinct roles in tissue injury and repair [40,41]. Similar phase-dependent immune responses may also influence HMGB1 signaling in renal IRI. Studies regarding upstream regulation of HMGB1 to prevent the release of this protein and its effect on inflammation and kidney function have been conducted. For instance, Zhan et al. found that grape seed proanthocyanidin extract (GSPE) significantly prevented inflammation, histological damage, and impairment of renal function in bilateral and unilateral renal I/R mouse models [13]. Mechanistically, GSPE inhibited the nucleocytoplasmic shuttling and extracellular release of HMGB1. Similar results have been observed for ethyl pyruvate (EP) [12]. In addition to blocking the translocation of HMGB1, other studies have attempted to interfere with the downstream signaling cascade initiated by HMGB1. According to Wu et al., treatment with rHMGB1 preconditioning decreased the inflammatory responses via TLR4 while upregulating the activity of Siglec-G, which suppresses immune stimulation [11]. This work highlights the complex signaling mechanism of HMGB1, which is potentially influenced by its redox status or distinct isoform configuration. However, using rHMGB1 alone could be limiting for translational purposes. Alongside these studies of HMGB1 antagonism, several natural compounds have also been investigated for their ability to modulate HMGB1 signaling. Lau et al. discovered that Glycyrrhizic acid (GZA) selectively inhibited HMGB1-driven cytokine secretion and NK cell activation [10]. Although promising, this work was not supplemented with pharmacokinetics and toxicity data. Importantly, the effects of HMGB1-targeted intervention have also been evaluated in a large-animal model. In this regard, Miura et al. demonstrated that peri-reperfusion administration of an HMGB1-neutralizing antibody in miniature swine reduced circulating HMGB1 levels, renal dysfunction, tubular injury, and apoptosis, providing large-animal evidence for the renoprotective effects of HMGB1 neutralization [9]. Despite the promising results, species-specific immune differences in swine models may limit the extrapolation of these findings to human applications. Collectively, these findings indicate the multifaceted role of HMGB1 in initiating and sustaining renal inflammation following IRI. Its dual capacity as a nuclear stress sensor and extracellular immune activator provides a mechanistic rationale for further investigation of HMGB1 as a therapeutic target.

Receptor usage is likely context-dependent rather than redundant. TLR4-MD-2 preferentially recognizes disulfide HMGB1 and is most strongly linked to rapid MyD88/NF-κB/MAPK cytokine signaling during acute reperfusion, whereas RAGE promotes HMGB1 internalization, sustained signaling, endothelial permeability, and later profibrotic responses. Both receptors can be co-expressed and co-activated, and TLR2 may provide additional inflammatory signaling. Renal studies support TLR4 dominance in several acute rodent models and RAGE involvement in ER stress and AKI-to-fibrosis, but direct head-to-head receptor blockade across matched time points is lacking. The available evidence therefore favors partially overlapping, phase- and cell-dependent functions rather than a fixed sequential hierarchy [18,26,35,37,42,43].

### 4.2. HMGB1 and Oxidative Stress

Besides an established role in mediating inflammation, HMGB1 is emerging as a key player in mediating oxidative stress in IRI [34]. Oxidative stress occurs when there is an imbalance between the formation of ROS and the antioxidant system, resulting in cell damage and dysfunction [44]. In this regard, Zhou et al. showed that xanthine oxidase-mediated ROS production increases HMGB1 expression, thereby forming a ROS–HMGB1 positive feedback loop that amplifies oxidative stress [8]. Allopurinol, a xanthine oxidase inhibitor, indirectly reduced HMGB1 expression by suppressing oxidative stress, as reflected by decreased MPO levels and restored SOD activity. While the above study provides crucial information, more research is needed to fully understand the implications of indirect HMGB1 modulation, including its effect on renal tissue repair and regeneration. To expand on this topic, some studies have explored the role of other factors that target pathways involved in oxidative stress downstream of HMGB1. The adipokine VASPIN was shown to reduce oxidative stress by activating the nuclear factor erythroid 2-related factor 2 (Nrf2)/antioxidant response element (ARE)/heme oxygenase-1 (HO-1) signaling pathway while suppressing HMGB1 expression [15]. Similarly, pentoxifylline (PTX) has also been reported to inhibit HMGB1 mediated oxidative stress and apoptosis [16]. Nonetheless, the absence of assessment regarding the optimum dosing protocols and possible adverse effects on the body is an important flaw. Notably, a number of recent studies have shed light on the involvement of the HMGB1-dependent oxidative stress in the development of chronic kidney disease (CKD) after AKI. Thus, Zhao et al. demonstrated that HMGB1 signaling activation predisposes tubular cells to oxidative stress, resulting in accelerated CKD development [17]. This long-term view allows us to understand better the pathophysiological significance of the involvement of HMGB1 in renal damage during the inflammatory process. Collectively, these studies underscore the complex interplay between HMGB1 and oxidative stress in renal IRI and support further investigation of this axis as a potential therapeutic target.

### 4.3. HMGB1 and ER Stress

HMGB1 also plays an important role in the pathogenesis of renal injury via regulating ER stress. ER stress occurs due to the inability of the ER to cope with the demand for protein folding, thus causing the activation of unfolded protein response (UPR), which ultimately leads to the induction of apoptosis [45]. In a model of intestinal I/R-induced remote acute kidney injury, Lai et al. revealed that HMGB1 signaling promoted ER stress and tubular cell apoptosis by activating TLR4 and RAGE signaling pathways [18]. Pharmacological inhibition of HMGB1 by ethyl pyruvate and anti-HMGB1 antibodies significantly attenuated the occurrence of ER stress and improved the outcome of renal diseases. While the above data present convincing evidence about the relationship between ER stress and HMGB1, further investigation in different kidney damage models should be carried out to determine whether the above therapeutic strategy can be applied in clinical settings. As a complementary approach, another adipokine named VASPIN has been found to decrease ER stress via downregulating HMGB1 expression, thus maintaining cellular homeostasis [15]. Although VASPIN has shown protective effects in preclinical models, the limited in vivo evidence precludes conclusions regarding its therapeutic applicability. Together, these studies support a multifaceted contribution of HMGB1 to ER stress-mediated renal injury and provide a rationale for further mechanistic and therapeutic investigation.

### 4.4. HMGB1 and Apoptotic Pathways

HMGB1 plays a role in renal damage through apoptosis induction via the intrinsic and extrinsic pathways. The lncRNA MEG3 was described by Mao et al. as an upstream factor that aggravates apoptosis by inhibiting the expression of miR-129-5p, a direct antagonist of HMGB1 [19]. Importantly, this study emphasized that HMGB1-mediated apoptosis can be modulated by non-coding RNAs. Notably, this work did not consider any variations of the regulatory mechanism according to the type of renal damage. Several pharmacological interventions have attenuated HMGB1-associated apoptosis in preclinical models. For instance, liraglutide, a glucagon-like peptide-1 (GLP-1) receptor agonist, inhibits the nuclear-to-cytoplasmic translocation and extracellular release of HMGB1, suppresses caspase-3 activation, and reduces apoptosis in renal tubular epithelial cells [20]. Although these findings support further investigation of liraglutide in renal IRI, additional studies are required to determine the efficiency of its application in chronic damage models and the stability of effects. Moreover, the HMGB1-TLR4-IL-23-IL-17A signaling axis promotes neutrophil-mediated apoptosis in renal tissue [42], underscoring the interplay between inflammatory and apoptotic pathways. These results offer important insights regarding mechanisms but require additional studies to determine the applicability of this approach in the clinical setting. Together, these studies emphasize the multifaceted involvement of HMGB1 in apoptosis during renal IRI and identify several mechanistically relevant targets that warrant further therapeutic investigation.

### 4.5. Integrated Signaling Network and Regulated Cell Death

Inflammation, oxidative stress, ER stress, and cell death are not parallel isolated modules. Extracellular HMGB1-TLR4/RAGE signaling activates NF-κB and MAPKs, increasing cytokines, leukocyte recruitment, xanthine oxidase activity, and ROS. ROS in turn promotes HMGB1 translocation and release and damages mitochondria and the ER, while PERK-ATF4-CHOP signaling and loss of mitochondrial membrane integrity converge on caspase-dependent apoptosis. Neutrophil recruitment through the HMGB1-TLR4-IL-23-IL-17A axis further increases ROS and protease burden. These feed-forward loops explain why interventions acting upstream at HMGB1 can affect several downstream endpoints simultaneously, but they also make it difficult to assign efficacy to one pathway from correlative measurements alone [8,16,18,20,42].

Beyond apoptosis, recent evidence directly links intracellular HMGB1 to ferroptosis. Cytoplasmic HMGB1 binds ACSL4 in tubular epithelial cells, and pharmacological blockade of HMGB1 nuclear export or tubular Hmgb1 deletion reduces lipid peroxidation, inflammation, and renal injury after I/R [46]. A recent study further connected glycolysis-derived lactate, HMGB1 lactylation, and cytoplasmic translocation to ferroptosis, with mild hypothermia interrupting this axis [47]. Necroptosis, pyroptosis, and PANoptosis are established components of renal IRI, but direct causal evidence placing HMGB1 upstream of these pathways in the kidney remains less developed. HMGB1 may be released as a consequence of lytic death and subsequently amplify neighboring inflammation; this bidirectionality should not be interpreted as proof that HMGB1 initiates every regulated cell-death program [4].

### 4.6. HMGB1 and Autophagy

HMGB1–autophagy interactions add another layer of context dependence. Under oxidative stress, cytoplasmic HMGB1 can bind Beclin-1, displace Bcl-2, activate ERK1/2, and sustain autophagic flux, thereby favoring cell survival over apoptosis [39]. Extracellular HMGB1 may also regulate autophagy through RAGE or TLR4, although the direction of effect varies with cell type and stimulus. Direct renal IRI evidence defining whether HMGB1-driven autophagy is protective, maladaptive, or phase-dependent is sparse. Therefore, inhibition of HMGB1 translocation could simultaneously reduce extracellular inflammation and ferroptosis while suppressing potentially adaptive Beclin-1-dependent autophagy; future studies should measure autophagic flux, rather than static LC3 or p62 abundance alone, and separate nuclear, cytoplasmic, and extracellular HMGB1 pools.

## 5. Therapeutic Strategies Targeting HMGB1

### 5.1. Direct HMGB1 Neutralization

Early investigations showed that neutralizing extracellular HMGB1 can attenuate the pro-inflammatory cascade and reduce renal damage. Specifically, Li et al. reported that treatment with HMGB1-neutralizing antibodies attenuated renal dysfunction and inflammation in a murine model [37]. Although this study provides preclinical evidence supporting this approach, it suffers from a short follow-up time and failure to examine chronic renal injury cases. Moreover, due to the lack of safety results, there might be an issue with immunogenicity when applying this approach in humans. Another study conducted by Miura et al. provided additional preclinical evidence that HMGB1 neutralization exerts protective effects in a large-animal model [9]. It showed that pretreatment of miniature swine with HMGB1-neutralizing antibodies improved renal functions and reduced apoptosis upon reperfusion. Despite the increased clinical relevancy of using miniature swine rather than rodents, the difference in immune responses between swine and humans could affect the effectiveness of such therapy. Moreover, the study does not address combinational treatment options or long-term renal improvement. Crucially, however, it has become clear that sex is an essential factor influencing the ability of neutralizing antibodies to counteract HMGB1 activity in IRI-induced renal damage. Indeed, Mohamed et al. demonstrated that spontaneous hypertensive rats (SHRs) undergoing IRI respond differently depending on their sex. Male SHRs experienced significantly higher levels of renal HMGB1 following IRI, compared to female SHRs. Additionally, these male rats experienced stronger inflammatory response and renal tubular cell damage than their counterparts [48]. The results of this study stress the importance of accounting for biological variables such as sex when developing therapies based on HMGB1-neutralizing antibodies. Alongside antibody treatment, another strategy worth considering includes the use of small-molecule inhibitors. For example, GZA functionally inhibits HMGB1-mediated inflammatory and cytotoxic responses and attenuates renal IRI [10]. Despite this promise, the pharmacological characteristics and toxicity of GZA need to be explored in further research. Furthermore, no adequate studies were conducted yet regarding the effect of GZA on preventing renal IRI in vivo in the long run. Collectively, these studies indicate the therapeutic potential of both antibody- and small-molecule based strategies to neutralize extracellular HMGB1 and mitigate renal inflammation. Nevertheless, key challenges remain, including defining the optimal therapeutic window, ensuring long-term safety, and validating efficacy across diverse kidney injury models.

### 5.2. Blocking HMGB1 Release or Translocation

Taking note of the importance of HMGB1 nucleocytoplasmic shuttling and extracellular release in eliciting pathogenesis, there have been attempts to inhibit these processes recently. For instance, Rabadi et al. demonstrated that EP inhibited HMGB1 translocation and release and attenuated renal injury after IRI [14]. EP treatment reduced acute renal dysfunction and inflammatory responses and also improved longer-term albuminuria and renal fibrosis in this model. Nevertheless, the optimal dosing regimen, pharmacokinetic and safety profiles, and reproducibility of these protective effects in clinically relevant models remain to be established. In another study, Ruan et al. showed that administration of carbon monoxide through carbon monoxide releasing molecule-2 (CORM-2) regulated histone acetylation and inhibited HMGB1 secretion to exert a protective effect against fatal renal IRI [49]. While the aforementioned results provided important information regarding the role of epigenetic regulation of HMGB1 as well as the cytoprotective role of carbon monoxide, the application of the latter may be restricted owing to CO toxicity in addition to a need for targeted delivery approaches. The studies failed to investigate whether carbon monoxide can prevent fibrosis or CKD in animal models. Most recently, Li et al. identified a connection between metabolic signaling pathways and the regulation of HMGB1, reporting that liraglutide blocks HMGB1 from leaving the nucleus of tubular epithelial cells [20]. This research expands on the scope of therapy options through its implication of metabolic pathways in controlling HMGB1. It should be noted that the potential therapeutic value of liraglutide in treating CKD has yet to be investigated, and off-target effects and drug interactions must be considered. Taken together, these findings highlight the potential utility of regulating the early stages of HMGB1 signaling to reduce damage to the kidneys. Nevertheless, the practical implications of these strategies call for more evidence from clinical trials.

### 5.3. Epigenetic Regulation

The emerging therapeutic approaches include epigenetic regulation of HMGB1 expression and function to prevent renal IRI. For instance, Zhang et al. found out that dexamethasone inhibits HMGB1 acetylation as well as translocation of HMGB1 to the cytosol via blocking the ERK/NF-κB signaling pathway [35], providing a mechanistic basis for the renoprotective effects of glucocorticoids. Although this information underscores the possible use of glucocorticoids to inhibit HMGB1 function, it should be noted that the detrimental long-term effects of using dexamethasone, including immunosuppressive effects, have not been fully addressed yet. In addition, Xu et al. showed that TUG1 knockdown increased miR-449b-5p and reduced HMGB1/MMP2 expression, thereby attenuating inflammation and apoptosis in renal tubular epithelial cells [36]. Mao et al. further demonstrated that MEG3 aggravated hypoxia/reoxygenation-induced tubular epithelial cell apoptosis through the miR-129-5p/HMGB1 axis [19]. Extending the above discussion, Lin et al. found that the regulation of HMGB1 via the axis involving the interaction between the lncRNA KCNQ1OT1 and miR-211–5p acted as an important epigenetic mechanism of HMGB1 control in renal IRI [50]. They found that administration of sufentanil, which is known as an analgesic opioid, down-regulated the expression of KCNQ1OT1 in rats modeling renal IRI, resulting in up-regulation of miR-211-5p. It was demonstrated that since miR-211–5p is a direct suppressor of HMGB1, a decreased level of cellular apoptosis, oxidative stress, and production of pro-inflammatory cytokines occurred. Overall, these findings suggest that RNA-based approaches may provide a potential strategy for modulating HMGB1, although their therapeutic applicability remains to be established.

### 5.4. Receptor-Specific Interventions

An alternative targeting HMGB1’s downstream receptors would provide a more focused strategy for minimizing harmful signaling outcomes of HMGB1 in renal IRI. Wu et al. proposed such an idea by demonstrating that preconditioning with recombinant HMGB1 increases Siglec-G expression, which negatively regulates TLR4-mediated inflammation [11]. This result highlights the complexities associated with HMGB1 signaling since HMGB1 isoforms or redox states could potentially exhibit opposing functions. Nevertheless, the use of recombinant HMGB1 in this experiment may not fully capture the nature of HMGB1 activity in vivo. In addition, the preconditioning approach described in this experiment may be impractical when applied to an acute clinical setting. To improve the receptor-based strategy, Zhang et al. showed that inhibition of the IL-23/IL-17A axis interferes with the HMGB1-TLR4-neutrophil axis, ultimately leading to decreased oxidative stress and apoptosis [42]. Although this approach showed protective effects in the experimental model, it might not fully address other potential inflammatory pathways that involve HMGB1 signaling. Additionally, the applicability of this strategy in the clinical setting still needs to be investigated. Moreover, angiotensin receptor blockers like azilsartan have also been reported to exert their action via blocking HMGB1-induced NF-κB and MAPK signaling cascade activation [51]. This opens up new ways regarding the ability of these molecules to manipulate HMGB1 pathways. Nevertheless, there is still a need to explore further if such drugs can affect patients undergoing multiple therapies simultaneously. In addition, further studies should examine the effect of azilsartan in combination with other anti-fibrotic agents such as SGLT2 inhibitors. More recently, Otsuka et al. investigated RAGE-related and mineralocorticoid receptor (MR)-targeted approaches that attenuated HMGB1-associated renal injury in preclinical models [43]. Although these findings provide preclinical support for an anti-fibrotic approach, there will be some difficulties in translating this approach into a clinical setting due to targeted drug delivery issues. Also, there might be other risks associated with MR antagonists such as hyperkalemia. Collectively, receptor-specific interventions offer a balance between broad anti-inflammatory efficacy and targeted pathway selectivity. Nonetheless, optimizing these therapies for clinical use remains challenging, especially considering polypharmacy and patient-specific factors that influence treatment responses.

### 5.5. Comparative Synthesis and Translational Readiness

Across strategy classes, direct neutralizing antibodies provide the clearest target specificity and have shown benefit when administered before ischemia in mice and immediately before reperfusion in miniature swine. Their strengths are mechanistic specificity and the ability to neutralize extracellular HMGB1 without directly suppressing nuclear functions; limitations include systemic exposure, tissue penetration, immunogenicity, manufacturing cost, and uncertain effects on host defense and repair. Small-molecule HMGB1 binders such as GZA are easier to formulate but have weaker target selectivity and incomplete pharmacokinetic and toxicology data. Inhibitors of HMGB1 acetylation, translocation, or release (EP, dexamethasone, CORM-2, liraglutide, and GSPE) act upstream and often suppress multiple injury pathways, but most were given prophylactically and have substantial off-target pharmacology. Receptor-directed strategies may preserve intracellular and CXCR4-mediated reparative functions, yet TLR4/RAGE overlap and cell-specific receptor expression may permit pathway escape. RNA and epigenetic strategies offer molecular precision but currently face renal delivery, stability, immunogenicity, and off-target barriers.

## 6. Concluding Remarks and Future Perspective

**Figure 1:**
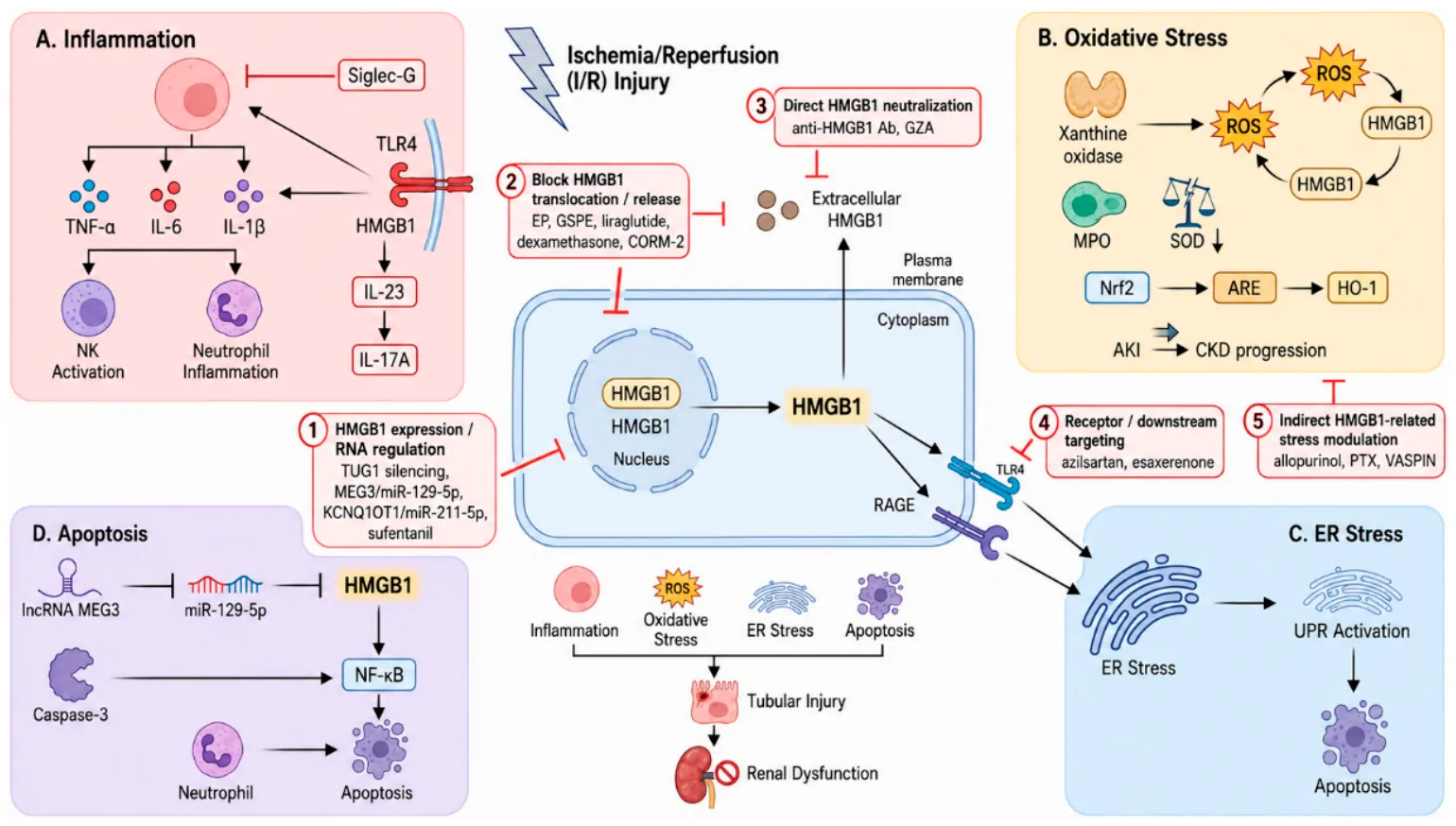
Schematic illustration of HMGB1-mediated mechanisms in renal ischemia–reperfusion injury (IRI). Following renal IRI, HMGB1 translocates from the nucleus to the extracellular space and activates TLR4 and RAGE, driving multiple injury pathways. (**A**) HMGB1 promotes inflammation by inducing TNF-α, IL-6, and IL-1β, leading to NK cell activation and neutrophil-mediated responses via the IL-23–IL-17A axis, while Siglec-G acts as a negative regulator. (**B**) HMGB1 amplifies oxidative stress through a ROS-dependent positive feedback loop, accompanied by MPO/SOD imbalance and impaired antioxidant defense involving the Nrf2/ARE/HO-1 pathway. (**C**) HMGB1 induces ER stress and UPR activation, resulting in apoptosis. (**D**) HMGB1 further promotes apoptosis via caspase-3, NF-κB signaling, and the MEG3/miR-129-5p axis. These pathways collectively lead to tubular injury and renal dysfunction.

Injury to the kidney after ischemia and reperfusion is still a medical problem since it results in AKI and subsequent development of CKD. As for the pathology involved in such a condition, the processes of inflammation, oxidative stress, and apoptosis play a significant role since they lead to the impairment of the functioning of tissues. Considering the variety of molecules that take part in this complex process, HMGB1 plays an important part as a mediator that organizes and enhances the inflammation leading to the kidney injury. This review summarizes the role of HMGB1 in renal IRI across inflammation, oxidative and ER stress, apoptosis and other regulated cell-death pathways, autophagy, and tissue repair (Figure 1 and Table 1). Nevertheless, there are still some interesting issues that require clarification.

### 6.1. Model Heterogeneity, Evidence Quality, and Publication Bias

Cross-study comparison is constrained by ischemia durations, unilateral versus bilateral clamping, prior nephrectomy, warm/cold ischemia combinations, different species and sexes, and follow-up periods ranging from hours to two months, although most studies focused on acute or short-term outcomes. A 30 min mouse model and a 120 min warm-ischemia swine model do not estimate the same biological severity, exposure window, or therapeutic effect. Most studies used small groups, male animals, surrogate biomarkers, and prophylactic dosing; few reported blinded outcome assessment, prespecified sample-size calculations, complete dose–response curves, PK/PD, toxicology, or long-term fibrosis. Outcome reporting is also inconsistent: some studies include survival, whereas others report creatinine, histology, or molecular markers without directly comparable effect sizes. Because the published record is dominated by positive studies and formal publication-bias analysis is impossible with heterogeneous designs, benefit is likely overestimated. The conclusions should therefore be interpreted as target validation and hypothesis generation, not proof of clinical efficacy.

### 6.2. Therapeutic Window, Safety, and Kidney-Directed Delivery

The optimal therapeutic window likely differs by clinical setting. Preconditioning is feasible in planned kidney transplantation or major vascular surgery, whereas unpredictable AKI requires treatment at reperfusion or shortly thereafter. Prophylactic administration has been commonly used in preclinical studies; for example, anti-HMGB1 antibody was administered before renal ischemia in mice [37]. However, post-reperfusion efficacy is not entirely unexplored. Wu et al. demonstrated that HMGB1-neutralizing antibody administered soon after ischemia-reperfusion also afforded significant renal protection in mice [38]. Nevertheless, post-reperfusion evidence remains limited and requires validation across clinically relevant models. Serial measurement of circulating or urinary HMGB1, redox isoforms, and renal injury biomarkers should be integrated into future studies to define when proinflammatory HMGB1 predominates and when repair begins.

Duration is as important as initiation. Because HMGB1 supports innate immunity, autophagy, and tissue repair, prolonged systemic inhibition could increase infection risk or impair epithelial regeneration. Future studies should compare short-pulse versus sustained blockade, early versus delayed treatment, and extracellular-selective versus total HMGB1 inhibition, with infection, wound healing, fibrosis, and CKD transition as safety outcomes.

Kidney-directed delivery may improve the therapeutic index. Cilastatin-functionalized nanoparticles have enabled proximal-tubule-targeted dexamethasone delivery and improved renal injury in murine AKI models, including bilateral renal I/R [52], whereas hypoxia-responsive nanoparticles have achieved renal tubular delivery of siCD36 in IRI-induced AKI [53]. These platforms have not yet validated HMGB1-selective cargo in renal IRI, but they provide a rational route for anti-HMGB1 antibodies or fragments, siRNA, antisense oligonucleotides, or CRISPR-based repression. Required development steps include biodistribution, renal-cell specificity, endosomal escape, immunogenicity, biodegradation, scalable manufacturing, and testing in large animals.

### 6.3. Combination and Clinical Trial Priorities

Combination therapy should be mechanism-based rather than additive by default. HMGB1 inhibition may be paired with standard nephroprotective measures or agents that target complementary nodes, including SGLT2 inhibitors, renin–angiotensin system blockade, antioxidant or ferroptosis-directed therapy, and ex vivo perfusion. However, combinations may also increase immunosuppression, hemodynamic effects, or off-target toxicity. A translational program should first identify a redox- and compartment-selective agent, establish PK/PD and a minimally effective exposure, confirm benefit in aged animals of both sexes and comorbidity models, validate efficacy in a large-animal transplant or surgical IRI model, and then proceed to biomarker-enriched phase I/II trials.

Overall, HMGB1 is a biologically compelling, pleiotropic regulator of renal IRI, but not yet a clinically validated therapeutic target. The most defensible near-term strategy is brief, early, extracellular- or receptor-selective modulation combined with kidney-directed delivery, rather than prolonged systemic suppression. Translation will require redox-resolved biomarker studies, rigorous comparative experiments, long-term repair and infection outcomes, and human PK and safety data.

## Figures and Tables

**Table 1:** Summary of HMGB1-targeted or HMGB1-related interventions in renal IRI and related acute kidney injury models.

Intervention	Model	Treatment Strategy	Outcomes	Mechanisms	References
HMGB1 neutralizing antibody	Mice subjected to 60 min ischemia followed by reperfusion	HMGB1 neutralizing antibody administered i.p. 24 h and 30 min prior to renal ischemia at a dose of 600 μg per mouse each time	Improved renal function Improved renal pathological damage	HMGB1 release ↓ TNF-α levels ↓ Tubular apoptosis ↓	[37]
HMGB1 neutralizing antibody	Swine subjected to 120 min warm ischemia followed by 60 min cold ischemia	1 mg/kg HMGB1 neutralizing antibody injected intravenously just before reperfusion of renal blood flow	Improved renal function Improved renal pathological damage	Serum HMGB1 levels ↓ Tubular epithelial apoptosis ↓ Neutrophil infiltration ↓ Tubular epithelial regeneration/proliferation ↑	[9]
HMGB1 neutralizing antibody	Rats subjected to 45 min ischemia followed by 24 h reperfusion	300 µg/rat HMGB1 neutralizing antibody i.p. injection 1 h prior to renal ischemia	Improved renal function in male rats Improved renal pathological damage in male rats	HMGB1-TLR4 ↓ TNF-α, IL-1β ↓ CCL2, CXCL1, CXCL2, CXCR1 ↓	[48]
HMGB1 neutralizing antibody	Mice subjected to 60 min ischemia followed by reperfusion	300 μg/mouse HMGB1 neutralizing antibody i.v. injection 1 h prior to renal ischemia	Improved renal function in mice Improved renal pathological damage in mice Improved survival rate	HMGB1 nuclear-cytoplasmic translocation and release ↓ TLR4, IL-23, IL-17A ↓ ROS, MDA ↓ SOD, CAT, GPX ↑ Tubular apoptosis ↓	[42]
GZA	Mice subjected to 45 min ischemia followed by reperfusion	1 mg GZA injected i.p. 2 h preoperatively and postoperatively at 8 h and 24 h after IRI.	Improved renal function Improved renal pathological damage	MCP-1, CXCL1, IL-6 ↓ NK cell activation ↓	[10]
EP	Rats subjected to 40 min of bilateral renal warm ischemia followed by reperfusion	Ringer’s EP solution administered at 100 mg/kg 20 min before ischemia and immediately after reperfusion	Improved renal function	HMGB1 release ↓ TNF-α ↓	[12]
EP	Mice subjected to 40 min ischemia followed by reperfusion	40 mg/kg EP i.p. injection 15 min prior to IRI surgery	Improved renal function	HMGB1 nuclear-cytoplasmic translocation and release ↓ TNF-α, IL-6, MCP-1 ↓	[14]
EP	Rats subjected to 1.5 h of superior mesenteric artery occlusion followed by 6 h of reperfusion	40 mg/kg EP i.v. injection 2 h before ischemia	Improved renal function	HMGB1 ↓ TLR4-RAGE ↓ PERK, ATF4, CHOP ↓	[18]
Liraglutide	Right nephrectomy, left renal pedicle clamped for 34 min, followed by reperfusion in mice	Six doses of liraglutide before ischemia (every 12 h from −60 to −12 h, −1 h) combined with 1 dose at 10 h after reperfusion, 200 μg/kg s.c. each time	100% survival rate Improved renal function Improved renal pathological damage	HMGB1 nuclear-cytoplasmic translocation and release ↓ HAT activity, HMGB1 acetylation ↓ Tubular cell apoptosis ↓	[20]
GSPE	Mice subjected to 30 min ischemia followed by 24 h reperfusion	150, 250, or 500 mg/kg GSPE orally for 5 days before ischemia	Improved renal function Improved renal pathological damage	HMGB1 nuclear-cytoplasmic translocation and release ↓ HMGB1-TLR4-NF-κB p65 ↓ TNF-α, IL-6, IL-1β,CCL2 ↓	[13]
Allopurinol	Rats subjected to 30 min ischemia followed by 72h reperfusion	50 mg/kg allopurinol administered i.p. for 14 days prior to ischemia	Improved renal function Improved renal pathological damage	HMGB1 ↓ Apoptosis ↓ MPO ↓ SOD ↑	[8]
PTX	Rats subjected to 45 min ischemia followed by 72 h reperfusion	PTX (100 mg/kg, orally) administered for 3 days after renal I/R	Improved renal function Improved renal pathological damage	ASK-1-JNK ↓ ERK-NF-κB p65-HMGB1 ↓ MDA ↓ TAC ↑	[16]
Azilsartan	Rats subjected to 30 min ischemia followed by 48 h reperfusion	Oral administration of 4 mg/kg azilsartan daily, starting 7 days prior to ischemia until the end of reperfusion (total 9 days)	Improved renal function Improved renal pathological damage	HMGB1-NF-κB ↓ IL-1β, IL-6, TNF-α ↓ GPX, SOD ↑	[51]
Sufentanil	Rats subjected to 45 min ischemia followed by reperfusion	1 mL sufentanil (2 μg/kg) injected 5 min before abdominal closure in I/R rats	Improved renal function	lncRNA KCNQ1OT1-miR-211–5p-HMGB1 ↓ TLR4-MyD88-NF-κB ↓ ROS ↓	[50]
Dexamethasone	Mice subjected to 1 h ischemia followed by 24 h reperfusion	4 mg/kg dexamethasone injected i.p. 1 h prior to ischemia	Improved renal function Improved renal pathological damage	HMGB1 nuclear-cytoplasmic translocation and release ↓ HMGB1 acetylation ↓ HMGB1-TLR4 ↓ ERK-NF-κB p65 ↓ TNF-α, IL-6, IL-1β ↓	[35]
Esaxerenone	Mice subjected to 22 min ischemia followed by 24 h reperfusion	3 mg/kg esaxerenone per day at 48, 24, and 1 h before I/R induction; Administered daily from 24 h after reperfusion	Improved renal function Improved renal pathological damage	HMGB1-RAGE ↓ Rac1 activation ↓ MR nuclear translocation ↓	[43]
VASPIN	Mice subjected to 40 min ischemia followed by reperfusion	Recombinant VASPIN was i.p. injected at 250 mg/(kg·d) for one week before constructing IRI model.	Improved renal function Improved renal pathological damage	HMGB1 ↓ IL-1β, TNF-α ↓ Nrf2/ARE/HO-1 ↑ MPO, MDA ↓ SOD, GSH-Px ↑ GRP78, ATF6, CHOP ↓	[15]
CORM-2	Mice subjected to 50 min left renal pedicle clamping followed by reperfusion	Single i.v. dose of 20 mg/kg CORM-2 1 h before renal ischemia	100% survival over 14 days Improved renal function Improved renal pathological damage	HMGB1 nuclear-cytoplasmic translocation and release ↓ TLR4, RAGE ↓ TNF-α, IL-1β, IL-6, MCP-1 ↓	[49]
Downregulation of lncRNA TUG1	OGD-induced injury in HK-2 cells	shRNA-TUG1-1 was transfected into HK-2 cells 48 h before OGD	Improved cell survival Decreased apoptosis	HMGB1, MMP2 ↓ miR-449b-5p ↑	[36]

Abbreviations: ↓: downregulation; ↑: upregulation; ARE: antioxidant response element; ASK-1: apoptosis signal-regulating kinase 1; ATF6: activating transcription factor 6; CAT: catalase; CHOP: CCAAT enhancer binding protein homologous protein; CCL2: chemokine (C-C motif) ligand 2; CXCL1: chemokine (C-X-C motif) ligand 1; CXCR1: C-X-C motif chemokine receptor 1; CORM-2: carbon monoxide-releasing molecule-2; ERK: extracellular signal-regulated kinase; EP: ethyl pyruvate; GRP78: glucose-regulated protein 78; GPX: glutathione peroxidase; GSH-Px: glutathione peroxidase; GSPE: grape seed proanthocyanidin extract; GZA: glycyrrhizic acid; IRI: ischemia-reperfusion injury; HAT: histone acetyltransferase; HK-2 cells: human renal proximal tubular epithelial cells; HMGB1: high mobility group box 1; HO-1: heme oxygenase-1; IL-1β: interleukin-1 beta; IL-6: interleukin-6; IL-17A: interleukin-17A; IL-23: interleukin-23; i.p.: intraperitoneal; i.v.: intravenous; JNK: c-Jun N-terminal kinase; KCNQ1OT1: potassium voltage-gated channel subfamily Q member 1 opposite strand/antisense transcript 1; lncRNA: long non-coding RNA; MyD88: myeloid differentiation primary response 88; MDA: malondialdehyde; MPO: myeloperoxidase; MMP2: matrix metalloproteinase-2; MR: mineralocorticoid receptor; NF-κB: nuclear factor kappa-B; Nrf2: nuclear factor erythroid 2-related factor 2; OGD: oxygen-glucose deprivation; PERK: protein kinase R-like endoplasmic reticulum kinase; RAGE: receptor for advanced glycation end products; ROS: reactive oxygen species; Rac1: Ras-related C3 botulinum toxin substrate 1; s.c.: subcutaneously; SMA: superior mesenteric artery; SOD: superoxide dismutase; TAC: total antioxidant capacity; TUG1: taurine upregulated gene 1; TNF-α: tumor necrosis factor-alpha; PTX: pentoxifylline; VASPIN: visceral adipose tissue-derived serine protease inhibitor; TLR4: Toll-like receptor 4.

## Data Availability

No new data were created or analyzed in this study. Data sharing is not applicable to this article.
